# Process model analysis of parenchyma sparing laparoscopic liver surgery to recognize surgical steps and predict impact of new technologies

**DOI:** 10.1007/s00464-023-10166-y

**Published:** 2023-06-29

**Authors:** Maryam Gholinejad, Bjørn Edwin, Ole Jakob Elle, Jenny Dankelman, Arjo J. Loeve

**Affiliations:** 1 Department of Biomechanical Engineering, Faculty of Mechanical, Maritime and Materials Engineering, Delft University of Technology, Delft, The Netherlands; 2 The Intervention Centre, Oslo University Hospital, Oslo, Norway; 3 Medical Faculty, Institute of Clinical Medicine, University of Oslo, Oslo, Norway; 4 Department of HPB Surgery, Oslo University Hospital, Oslo, Norway; 5 Department of Informatics, University of Oslo, Oslo, Norway

**Keywords:** Surgical process modeling, Discrete event simulation, Parenchyma sparing, Surgical step prediction, Surgical task recognition, Surgical data analysis, Surgical navigation platform

## Abstract

**Background:**

Surgical process model (SPM) analysis is a great means to predict the surgical steps in a procedure as well as to predict the potential impact of new technologies. Especially in complicated and high-volume treatments, such as parenchyma sparing laparoscopic liver resection (LLR), profound process knowledge is essential for enabling improving surgical quality and efficiency.

**Methods:**

Videos of thirteen parenchyma sparing LLR were analyzed to extract the duration and sequence of surgical steps according to the process model. The videos were categorized into three groups, based on the tumor locations. Next, a detailed discrete events simulation model (DESM) of LLR was built, based on the process model and the process data obtained from the endoscopic videos. Furthermore, the impact of using a navigation platform on the total duration of the LLR was studied with the simulation model by assessing three different scenarios: (i) no navigation platform, (ii) conservative positive effect, and (iii) optimistic positive effect.

**Results:**

The possible variations of sequences of surgical steps in performing parenchyma sparing depending on the tumor locations were established. The statistically most probable chain of surgical steps was predicted, which could be used to improve parenchyma sparing surgeries. In all three categories (i–iii) the treatment phase covered the major part (~ 40%) of the total procedure duration (bottleneck). The simulation results predict that a navigation platform could decrease the total surgery duration by up to 30%.

**Conclusion:**

This study showed a DESM based on the analysis of steps during surgical procedures can be used to predict the impact of new technology. SPMs can be used to detect, e.g., the most probable workflow paths which enables predicting next surgical steps, improving surgical training systems, and analyzing surgical performance. Moreover, it provides insight into the points for improvement and bottlenecks in the surgical process.

Improvement of surgeries is an ongoing challenge for researchers that can be achieved by providing new technological advancements, guiding surgeons during operation by prediction of next surgical steps, finding and dealing with surgical bottlenecks, improving surgeon’s training, etc. To obtain these goals, various disciplines need to work together to provide the right inputs for working on different aspects involved in improving surgical procedures. Surgical Process Models (SPMs) can be used to find the structural coherence of complex surgical procedures and for obtaining profound qualitative and quantitative understanding of the relations within the surgical procedure, its variation parameters, and its output parameters [1].

Predicting surgical steps, their sequence, and durations can aid the improvement of operations by supporting surgeons in their needs at the right moment and by monitoring the time management of surgery. Surgeons can use the predictions and the suggested probable sequence of surgical steps to perform efficient pre-operative planning as well as intra-operative treatment. Monitoring could be specifically helpful for the centralized management of personnel for efficient operation scheduling and resource management [2]. In addition, such predictions can be used to train the young generation of surgeons according to the most probable sequence of surgical steps. Aiming to address the aforementioned challenges, several attempts have been made in previous studies to first establish the surgical steps (either by manual annotation of an observer [3, 4] or using digital data in the OR [5, 6]) and then try to predict the sequence of the steps [7–9]. Several methods have been proposed for establishing the surgical steps from digital data (e.g., sensor and camera) in the OR, such as using hidden Markov model [10, 11], support vector machine classifiers [12], forest trees [13], and random forests [14]. Various data sources, such as anesthesia and vital sign data [15, 16], OR and endoscopic videos [13, 17, 18], signals from surgical robots [19], tool/device usage [20], and workflow recognition sensors [10, 21], have been used for intra-operative task discovery and predictions. Although several studies on revealing the surgical steps in a procedure are available in the literature, there are only few studies on the intra-operative prediction of successive steps [7–9]. Up to now, most of the prediction studies are focused on risk prediction models [22, 23], prediction of total operation duration [2, 24], and post-operative complications prediction [25–27]. Surgical operations are characterized by their highly variable process and duration. In this type of analysis, it is important to have the information at high level of detail (fine granularity level). Prediction of fine granularity surgical steps is challenging due to difficulties of recognizing surgical tasks, modeling of the highly variable surgical procedures and merging these highly varying procedures in order to determine the possible sequence of surgical steps [28]. Surgemes and dexems are the structure of SPMs in fine granularity levels. Surgemes are surgical steps and are defined as the entire act of performing a certain surgical task, while dexemes are the way of performing a surgical step at a lower level of abstraction or finer level of granularity [29].

Aim of this paper is to discover possible sequences and durations of surgical steps with a high level of details (fine granularity) for resection of different segments in parenchyma sparing, minimally invasive liver treatment (MILT) that will be used in predicting surgical steps, surgery durations, and predicting impacts of new technologies. By merging sets of individual Surgical Process Models (iSPMs) and an extensive statistical analysis of clinical data, we discover the (most) possible sequences of surgical steps and surgery duration. These sequences/steps are used to build a discrete event simulation model which is then used to predict the effects a novel navigation platform, prior to actual implementation into clinical practice [28]. In this study, we simulated a technologically feasible navigation platform; however, the methodology can be used for assessment of different new technologies.

## Method

### Data acquisition

Process data were acquired from endoscopic videos of thirteen parenchyma sparing of laparoscopic liver resection (LLR) for colorectal liver metastasis performed at Oslo University Hospital, Norway (OUH). All lesions were located in the right lobe of liver. To limit the variance in process data, only surgeries treating a single lesion located in one or two neighboring liver segments were included. Based on the segments that were being treated, the videos were categorized into three groups: (i) five videos of Segments 5&6 (no gallbladder removal), (ii) five videos of Segments 7&8, and (iii) three videos of Segment 5 (with gallbladder removal cholecystectomy). All the surgeries were performed at The Intervention Center of OUH by four different highly experienced lead surgeons with more than 10 years of surgical experience. After making observations in the operating room and conducting interviews with surgeons at Rikshospitalet, it was found that the surgeons generally employed similar surgical instruments and techniques. Ethical approval for this study was obtained from OUH in which the data were collected (Regional Ethical Committee of South Eastern Norway-REK Sør-Øst B 2011/1285 and the Data Protection Officer of OUH).

### Data analysis

**Fig. 1:**
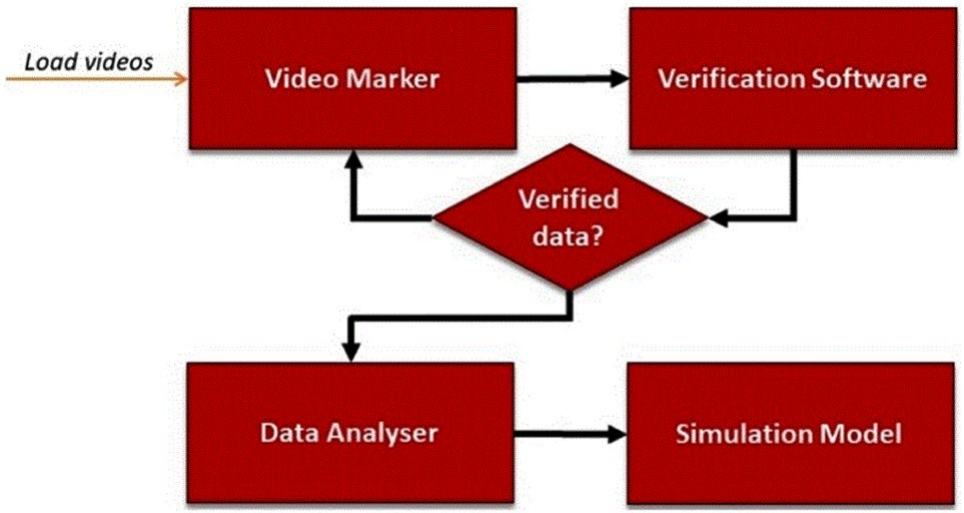
Integrated system for data analysis consisting of the software modules Video Marker, Verification Software, Data Analyser, and Simulation Model

The endoscopic videos of the surgeries were analyzed in order to divide the surgical procedures into surgical steps and to extract the sequence and durations of these steps. The surgical procedures were analyzed based on the generic surgical process model (GSPM) of MILT established in our previous work [30]. The duration, number of occurrence, and sequence of each surgical step were obtained by analysis of the endoscopic videos. An integrated in-house built software system was developed for registration, storage, verification, analysis, and simulation of surgical process data. Figure 1 shows a schematic of the modules in the developed system. The “Video Marker” module (developed in C# language) enables registration of the sequence, start time and end time of surgical steps on endoscopic videos. Next, the registered surgical data was verified using the “Verification Software” (developed in Unity engine, C#, and Java). The registered data and relevant registered information for each surgical step were visualized as a layer put over the generic MILT process model to confirm the flow of the registered surgery process. After confirmation, the surgical data were stored and analyzed in “Data Analyser” (programmed in Matlab). The results of the data analysis were fed into the Simulation Model of LLR to enable investigation of and judge the impact of introducing potential new technologies on the process and duration of LLR.

The surgical steps were numbered by phase and module numbers according to the process model in [30], see also Appendix. For example, “P07M01” refers to Phase 07, Module 01; Trocars Placement. For registration of the occurrence frequency of modules, as long as successive actions or decisions were in the same module, this was counted as a single occurrence. Five of the process model phases and modules were treated in particular ways during data registration and analysis for specific reasons:

- Intra-operative planning (P06): The surgeons generate/update the surgical plan while taking ultrasound (US) images. Consequently, in case of taking intra-operative images, imaging (P05M02) and planning (P06M01) run in parallel. During surgery, surgeons often have moments where they need to decide on their next course of action. These moments are typically very brief, usually lasting just a few seconds. As a result, these moments were not included as a separate planning step (planning module) in the surgical process, but instead were incorporated into the duration of the subsequent step. Planning module, on the other hand, refers to moments when the surgeon is examining and investigating the treatment region and surroundings through the endoscopic camera view. These planning moments can occur at any time during the surgery. Certain surgical tasks, like placing a trocar, always require planning, but the planning process is typically very rapid and may occur outside of the endoscopic camera view. In such cases, a zero duration was assigned to the planning step.
- Supply ducts division (P08aM06): In the GSPM definition, supply ducts (we also refer to them as supply vessels) include hepatic artery, portal vein, bile duct, and hepatic veins. Small vessels can be divided along with the action of resecting a section of the liver. However, larger branches of supply ducts, which require more attention, have to be isolated, (possibly) occluded and then divided by the surgeon (Modules 03, 05, and 06 in Phase 08a). An action is considered to be “Supply ducts division” when the vessel is distinguishable in the video, the surgeon isolates the vessels and divides them, regardless of whether permanent occlusion of the vessels is performed or not. Permanent occlusion is considered when the surgeon uses a clip or stapling device to occlude supply ducts. It should be noted that in parenchyma sparing, the modules involving supply duct division (Modules 03, 05, and 06 in Phase 08a) can be considered as part of the treatment phase (P10M02). However, we chose not to combine supply duct division with the treatment phase in our study to allow for a separate analysis of supply duct division. If necessary, these modules can be easily combined back into the treatment phase.
- Leakage clean-up (P11M02 and P13M04): The activities in this phase were separated in two intra-operative phases: during treatment of lesion as intra-operative complications (phase 12) and in wrap-up activities (phase 13). This is because leakage clean-up prior to completing treating a lesion can happen anytime during surgery, whereas leakage clean-up in wrap-up activities is part of a normal procedure and not a complication.
- Intra-operative preparation (P04): Intra-operative preparation (P03) typically takes about an hour and does not depend on tumor size, location, etc. Therefore, this duration was taken as a fixed time for this phase.
- Wrap-up activities (P13): During wrap-up activities (P13) some modules occur after the endoscopic camera has been taken out of the abdomen, therefore, no timings are recorded for these modules (e.g., exsufflation and incision closing-P13M08 and P13M09).

Note that there are moments in laparoscopic surgeries that are considered as Idle time, such as when the surgeon takes out the camera to clean the lens or when the surgeon is not performing any activities visible in the endoscopic video.

### Discrete event simulation

Following the approach of Loeve et.al. [28], to study the impacts of new technologies on LLR, a detailed discrete events simulation model of LLR was built in Matlab, based on the process model and the process data obtained from the endoscopic videos. Process model consists of modules and questions.

Modules: Due to the limited number of data points for each module (between 2 and 30), finding the true distribution of modules duration is elusive. The simulations were ran for two diverse distributions: Gaussian distribution and Uniform distribution. In the case of Gaussian distribution, for each module the probability distribution of its duration was calculated by fitting a Gaussian distribution function to the data obtained from the endoscopic videos. The negative tail of the Gaussian distribution was ignored in the simulation model, i.e., the duration probability distributions had a finite lower bound of zero. This means that the final result is a skewed non-symmetrical distribution rather than an actual Gaussian distribution. In the case of Uniform distribution, a random duration between shortest and longest duration of each module was generated.
Questions: It was assumed that the question outputs were instant, thus question durations were set to zero. The questions were defined as dynamic points in the simulated model. This means that the probabilities of the question outcomes depended on the number of times that question had already been executed.

Phase 11 of the process model can happen any time during a procedure. Implementation of these phases in the simulation is a tedious task. In order to prevent unnecessary complications in the simulations, the average duration of leakage clean-up (P11M02) were added to the simulations. Note that leakage clean-up is not affected by the introduction of navigation platform in the defined scenarios in the following section, thus it is safe to add the average duration of P11M02 to the simulations. Similar to P11M02, the average duration of Idle time was added to the simulations.

### Prediction of impact of new navigation platform

To analyze the effects of new technologies in LLR, we simulated a technologically feasible, new platform that improves visualization of the treatment area for the surgeons by showing a 3D model of the patient’s liver and internal structures, including vessels and supply ducts, such as the navigation platform being developed in the HiPerNav project [31, 32] (see also the video in https://www.youtube.com/watch?v=ix2bDXfQ0tc&t=9s). The platform visualizes the position of surgical instruments with respect to internal structures in the 3D model during operation or a visualization of internal structures as an overlay on the laparoscopic video (Augmented Reality). Based on the planned data, the platform guides the surgeons in performing surgical steps during the treatment. In these platforms, the medical images are obtained using different image modalities and a 3D model of the liver is generated based on these images. The described technology is the new technology that we assess its impact on the LLR in this work. Typically, image to image registration, image segmentation, and image-to-patient registration are required in these technologies [33–37]. On the other hand, navigation platforms facilitate several different steps in performing LLR. In order to predict the effects of such a navigation platform on liver surgery, the following three scenarios were defined.

Scenario 1: No use of the navigation platform.
Scenario 2: Navigation platform in use—conservative positive effect. The navigation platform has an impact on various modules, including Resection (P10M02), Marking (P10M01), Supply Duct Isolation (P08aM03), Planning (P06M01), and Imaging (P05). Enhanced visualization of patient’s organ and improving surgeon’s insight on positions of supply ducts, results in performing resection (P10M02) faster than normal. Therefore, the resection (P10M02) time was assumed to decrease by 10%. Additionally, the platform eliminates the need for physical marking of the resection area (P10M01), as it displays tumor borders and suggests the treatment margin in a 3D view. Supply duct isolation (P08aM03) is also simplified due to the known positions of the supply ducts, reducing the duration of this module by 25%. However, the platform adds some computational burden. The time for segmentation was set to 60s due to computational times. Image to 3D model registration will be done to update the 3D model, for which a computational time of 120s was implemented. For taking new images as the input for updating the model 120s was implemented. These technical durations were estimated based on the authors’ hands-on experience with available navigation systems [38, 39]. It was assumed that the image-to-patient registration is done prior to start of the surgery. During a surgical procedure, patient positioning might change. In the case of minor adjustments, no additional action is required. However, if significant changes occur, new images must be obtained, and the image-to-patient and 3D model registration processes, as well as image segmentation, must be repeated. For such cases, the additional time should be added to the total surgery time. We assumed no significant changes in patient positioning; however, recent studies have demonstrated that the process of updating the 3D model to account for the deformation of liver shape during surgery, can be accomplished more efficiently using surface reconstruction from intra-operative stereo video. These methods eliminate the need for the time-consuming and resource-intensive approaches that are considered in the scenarios 2 and 3 [40–42].
Scenario 3: Navigation platform in use—optimistic positive effect. For this scenario more optimistic performance was assumed. The resection (P10M02) time was decreased by 20% instead of 10%. Isolation of supply ducts (P08aM03) is taken to be faster, decreasing the duration of “Supply ducts isolation” by 50% instead of 25%. The time for updating the segmentation was put to 30s instead of 60s and image to 3D registration duration was halved to 60s.

The simulation is designed based on probability distribution functions for all modules occurrences and durations; therefore, criteria for excluding non-logical occurrences are defined. The exclusion criteria are (1) no less than 3 trocars are ever used and (2) the minimum time for resection is half of the minimum resection time observed in video data of that surgery category. The minimum times of resection in the real dataset available for Segments 5 & 6 was 476 s (approx. 8 min), for Segments 7 & 8, this was 540 s (9 min), and for Segment 5 with gallbladder removal, this was 922 s (approx. 15 min). Based on our previous observations in LLR, the resection in the surgeries with the minimum values could have been perform faster than the current total duration; thus, we have taken the half of these minimum values as the lower bound. It was necessary to define a lower bound for the duration of resection, as otherwise unreasonably small values (down to zero) could be allocated to the resection time in the simulations. The operational behavior of the simulation model was checked by observing the animated output of the simulation, which confirmed that it is comparable to the real data acquired from endoscopic videos.

## Results

Median lesion length in the laparoscopic surgeries that were analyzed was 75 mm (range 40–115 mm). All tumors were malignant, except for one. Median patient age was 66 (range 47–80) and 58% were male. Nine patients had prior abdominal surgery; 3 patients on the liver, out of which 2 patients previously underwent open liver resections. Three patients received neoadjuvant chemotherapy (NEO) prior to hepatic resection.

**Fig. 2:**
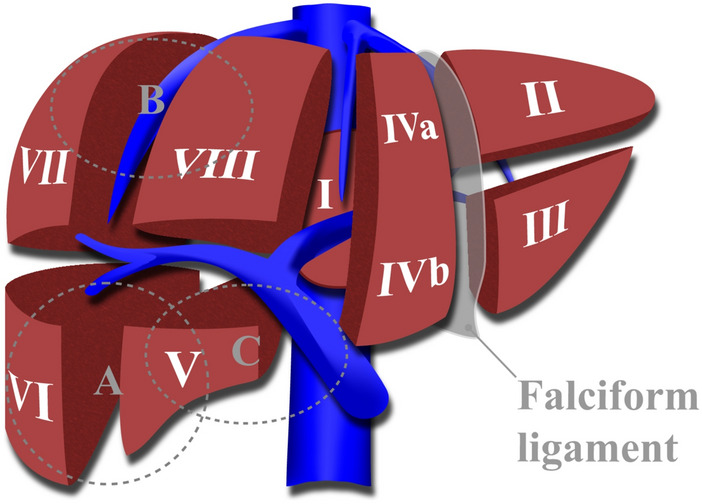
Eight functionally independent segments of liver. A, B, and C show the resection lesion margins of segments 5 & 6, 7 & 8, and 5 with gallbladder, respectively

Figure 2 shows the approximate resected lesion margins of the laparoscopic surgeries that were analyzed in this paper. It is worth mentioning that some procedures like cholecystectomy (Segment 5 with gallbladder removal) are more consistent and standardized. In the following paragraphs, while we discuss the different procedures for different segments, one can also see how the results altered for the steps of cholecystectomy (Segment 5 with gallbladder removal) with compared to Segments 5 & 6 (without gallbladder removal).

The sequence of surgical steps and the registered time per steps for all surgeries are given in https://doi.org/10.4121/20163968 and Ref. [30]. Table 1 shows the mean durations and occurrence frequencies of phases and modules for the three categories of parenchyma sparing surgeries extracted from the analyzed videos. Not all the phases and modules in the generic surgical process model of MILT occur in LLR, as this model also covers MILT variants. To interpret the data in Table 1, one needs to note that some modules do not occur in all surgeries (e.g., P08aM01); therefore, the average occurrence frequency can be less than 1. The standard deviation of occurrence frequency is zero for several modules indicating that the occurrence frequency values are the same for that module (e.g., P07M01). The mean duration of each module is calculated as the mean duration of that module in all surgeries, excluding those surgeries in which this module did not occur.

**Table 1:** The duration (in seconds) and occurrence frequency (number of occurrence) of the modules extracted from the endoscopic videos, presented as “mean (standard deviation)”

		Segments 5 & 6		Segments 7 & 8		Segment 5 (with gallbladder removal)	
Phase	Module	Duration (s)	Occurrence (-)	Duration (s)	Occurrence (-)	Duration (s)	Occurrence (-)
Imaging *(05)*	*2*	252 (188)	2 (1.2)	1259 (1491)	3.4 (1.3)	235 (184)	1 (1)
Planning *(06)*	*1*	85 (47)	5 (1.2)	181 (146)	7.8 (2.9)	222 (146)	6 (1.7)
Operative field access *(07)*	*1*	157 (87)	3.6 (0.5)	132 (73)	4 (0.7)	170 (40)	4 (0)
	*2*	N.R	1	N.R	1	N.R	1
Destructive Isolation* (8a)*	*1*	90 (0)	0.2 (0.4)	2692 (0)	0.6 (1.3)	209 (0)	0.7 (1.1)
	*2*	548 (43)	0.6 (0.9)	191 (10)	0.4 (0.5)	855 (414)	4 (1.7)
	*3*	400 (370)	5.6 (8.8)	0 (0)	0 (0)	150 (111)	1.33 (0.6)
	5	134 (120)	1.6 (2)	0 (0)	0 (0)	60 (26)	1.67 (0.6)
	6	410 (387)	5.4 (8.4)	0 (0)	0 (0)	38 (10)	1.67 (0.6)
Treatment *(10)*	*1*	176 (86)	1 (0)	197 (85)	1.2 (0.4)	259 (93)	1 (0)
	*2*	1343 (951)	2.6 (1.8)	1421 (736)	1.6 (0.9)	1210 (253)	1.7 (0.6)
Intra-Operative Complications* (11)*	2	222(172)	6.4 (3.3)	291(190)	7 (4.4)	347(291)	8 (7)
Wrap-up *(13)*	*2*	101 (81)	1 (0)	63 (20)	1	38 (3)	1.3 (0.6)
	*3*	52 (47)	1 (0.5)	105 (32)	1.4 (0.9)	227 (205)	1.3 (0.6)
	*4*	223 (220)	3.4 (1.7)	225 (222)	4.8 (3.3)	128 (72)	2.7 (0.6)
	*5*	100 (17)	1 (1.4)	99 (75)	1.2 (1.6)	75 (70)	0.7 (0.6)
	*6*	252 (120)	3 (1.2)	218 (144)	2.4 (2.9)	438 (340)	1.7 (0.6)
	*7*	0 (0)	0 (0)	98 (9)	0.8 (0.8)	0 (0)	0 (0)
Idle		193 (67)	–	931(1154)	–	322 (51)	–

N.R.: The duration was Not Recognizable in the videos

In the Imaging and Planning phases (P05 and P06) and Region Marking module (P0M01) in Table 1, while the surgeon is taking images using US (P05M02), planning is normally generated/updated. Hence, the total planning time is the sum of the imaging duration (P05) and the planning duration without imaging (P06), as shown in Table 1. Imaging activities (P05) can be done separately or in parallel with Region Marking in Phase 10. In case of parallel Region Marking-Imaging the timing and the occurrence frequencies are considered for both Imaging and Region Marking.

**Fig. 3:**
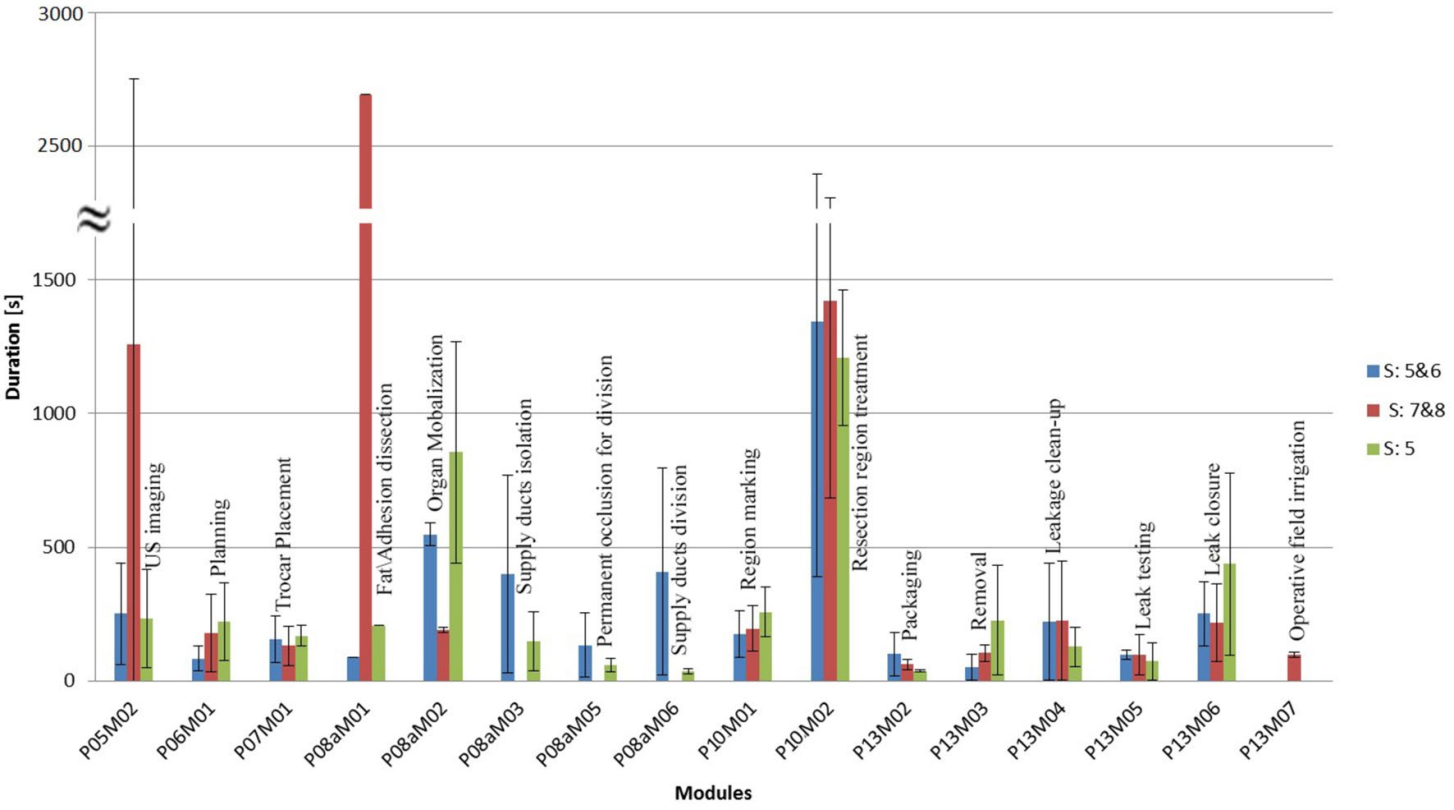
Mean duration (standard deviation indicated by whiskers) of the modules for each surgery category

**Fig. 4:**
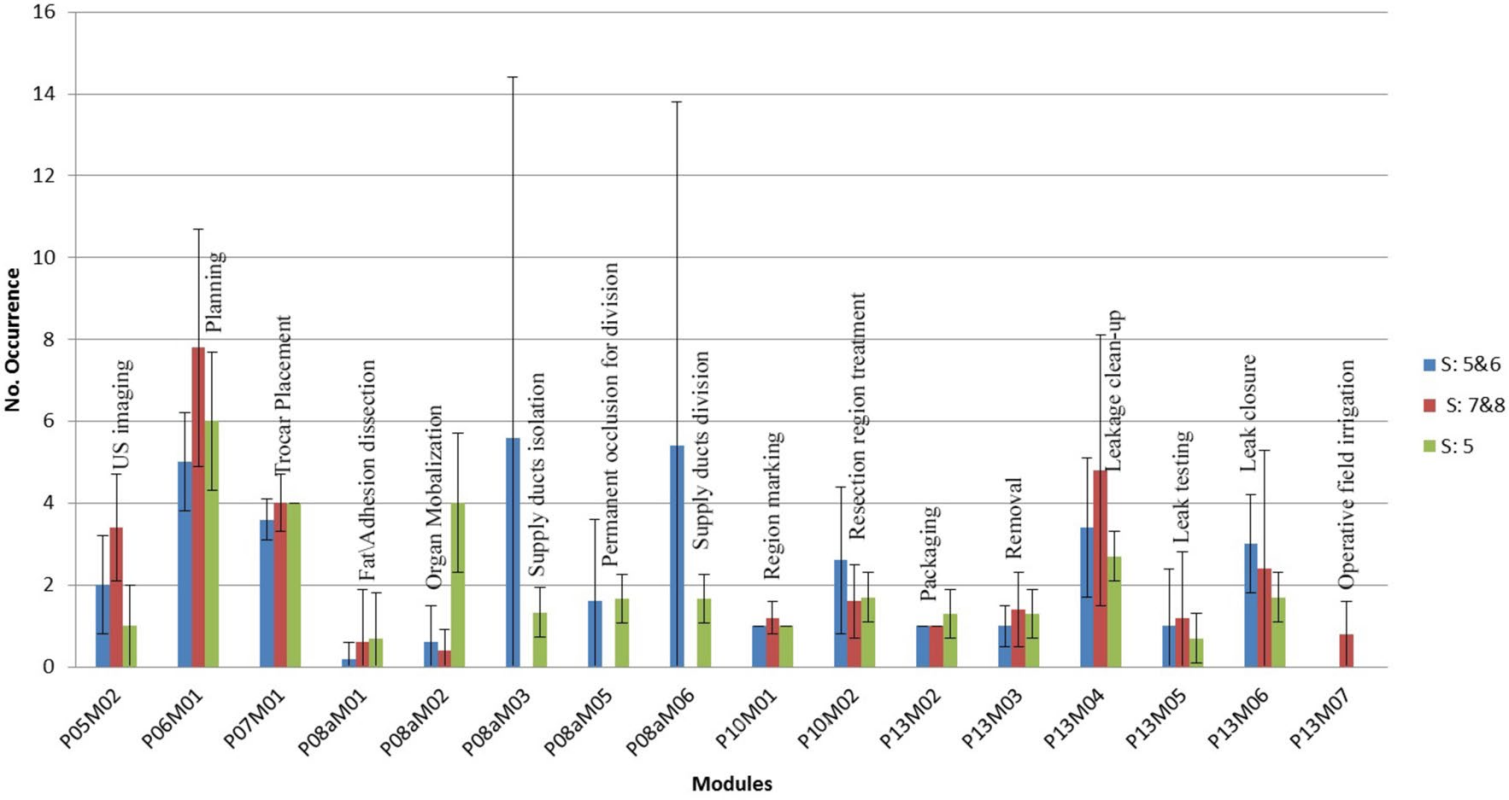
Mean occurrence frequency (standard deviation indicated by whiskers) of the modules for each surgery category

For an easy comparison of modules between different surgery categories, the duration and occurrence frequencies provided in Table 1, are also shown in Figs. 3 and 4, respectively.

For Segments 5 & 6 surgeries, 3.6 trocars were used on average compared to an average of 4 trocars for the two other categories. In Segment 5 with gallbladder removal, the surgeon always used 4 trocars, but in Segments 7 & 8, the surgeon used between 3 and 5 trocars. In the dataset analyzed for Segments 7 & 8, no instances of supply duct isolation (P08aM03), occlusion (P08aM05), or division (P08aM06) were observed. This is because although the right hepatic vein passes on the border of Segments 7 and 8 (and its branches penetrate into Segment 7) and a branch of the middle hepatic vein passes Segment 8, the tumors in our dataset were located in the upper parts of Segments 7 & 8. The occurrence frequency of supply duct isolation (P08aM03) was not equal to that of supply ducts division (P08aM06) for two reasons: (1) the surgeon isolated or divided more than one supply duct in a row in one occurrence of that module or (2) the surgeon performed other treatment activities after starting to perform isolation of a supply duct while the isolation is not yet completely done. The occurrence frequency of permanent occlusion (P08aM05) was less than isolation and division because not all the vessels require permanent closure before division. In case of Segments 5 & 6, an average of 1.6 vessels were occluded (with clips or Endo Gia stapler) out of 5.4 divided vessels. Surgeries in Segment 5, which involved gallbladder removal, always required occlusion of two supply ducts, leading to a longer duration for performing supply duct isolation (P08aM03), occlusion (P08aM05), or division (P08aM06) compared to other categories in the dataset. Surgeons typically used surgical clips to occlude the cystic ducts of the gallbladder. Although the permanent occlusion module had an occurrence frequency of less than 2 in Table 1, this was because the surgeon only occluded two supply ducts in a single instance of the module. As a result of the analysis, large differences in duration of the imaging module between resections of 7 & 8 segments and resections of 5 and 6 segments were observed. The result agrees with the fact that wedge resection of tumors in posterosuperior segments is difficult in laparoscopy due to the difficulties for access and poor visualization of these segments, thus assessing parenchyma structure and planning require more time of imaging.

The durations of the “fat/adhesion dissection” module (P08aM01) in Segments 5 & 6 and Segment 5 with gallbladder removal were considerably smaller than for Segments 7 & 8. However, the large duration differences of this module between different surgery categories are possibly due to patient condition rather than tumors location. Presence of fat or adhesions is known to be related to parameters, such as patient BMI, previous abdominal surgeries, and special diseases [43]. Treatment region marking (P10M01) was performed in all surgeries and with similar durations in all surgery categories (~ 200 s). Treatment (P10M2) durations were similar in different categories, but with different occurrence frequencies. The occurrence frequency of the treatment module in segment 5 & 6 surgeries was larger than for the other segments, as the surgeons had to take care of large branches of vessels while performing resection, resulting in more transitions in the flow between the treatment phase and the destructive isolation phase for performing supply ducts division during resection. Placement of new trocars and taking new images were other reasons for increasing occurrence of treatment module in different surgery categories. The duration and occurrence of wrap-up activates such as packaging (P13M02) and removal (P13M03) for removing resected tissue and un-absorbable materials, leakage clean-up (P13M04), and leak testing (P13M05) appeared to be not directly linked to the tumor location. Blood leakage volume and the size of the resected region are examples of influencing factors for durations and occurrences of the activities in this phase.

**Fig. 5:**
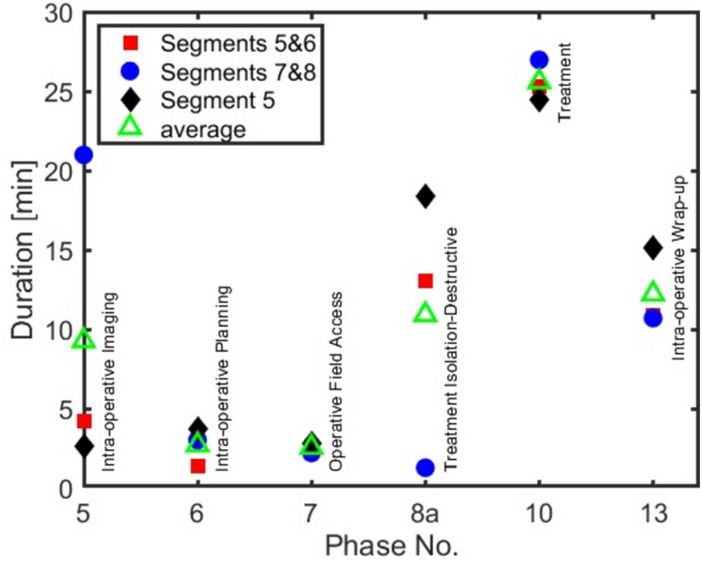
Average duration of each phase for different surgery categories. The open green symbols are the average of all three surgery categories. The phase names are given next to the symbols

Figure 5 shows the duration of each phase normalized to the sum of durations of all phases (which is the procedure duration). In this figure, the fat/adhesion dissection module (P8aM01) was excluded, as its duration was highly influenced by other factors, such as BMI and previous abdominal surgeries, more so than all other modules. In all three surgery categories, the surgeons spent most of their time on the treatment phase (P10); approximately 25 min (40% of total surgery time) and almost 85% of the treatment phase duration was allocated to the resection. In parenchyma sparing, supply ducts isolation and division may be considered as parts of resection, further increasing the dominance of the treatment phase. Destructive isolation (P08a) and wrap-up activities (P13) each took on average about 13 min (20%) of the surgery time. Imaging (P05) took on average less than 10 min (15%), while planning (P06) itself (without imaging) and making the operative field accessible (P07) each consumed less than 5 min (5%) of the total surgery time.

**Fig. 6:**
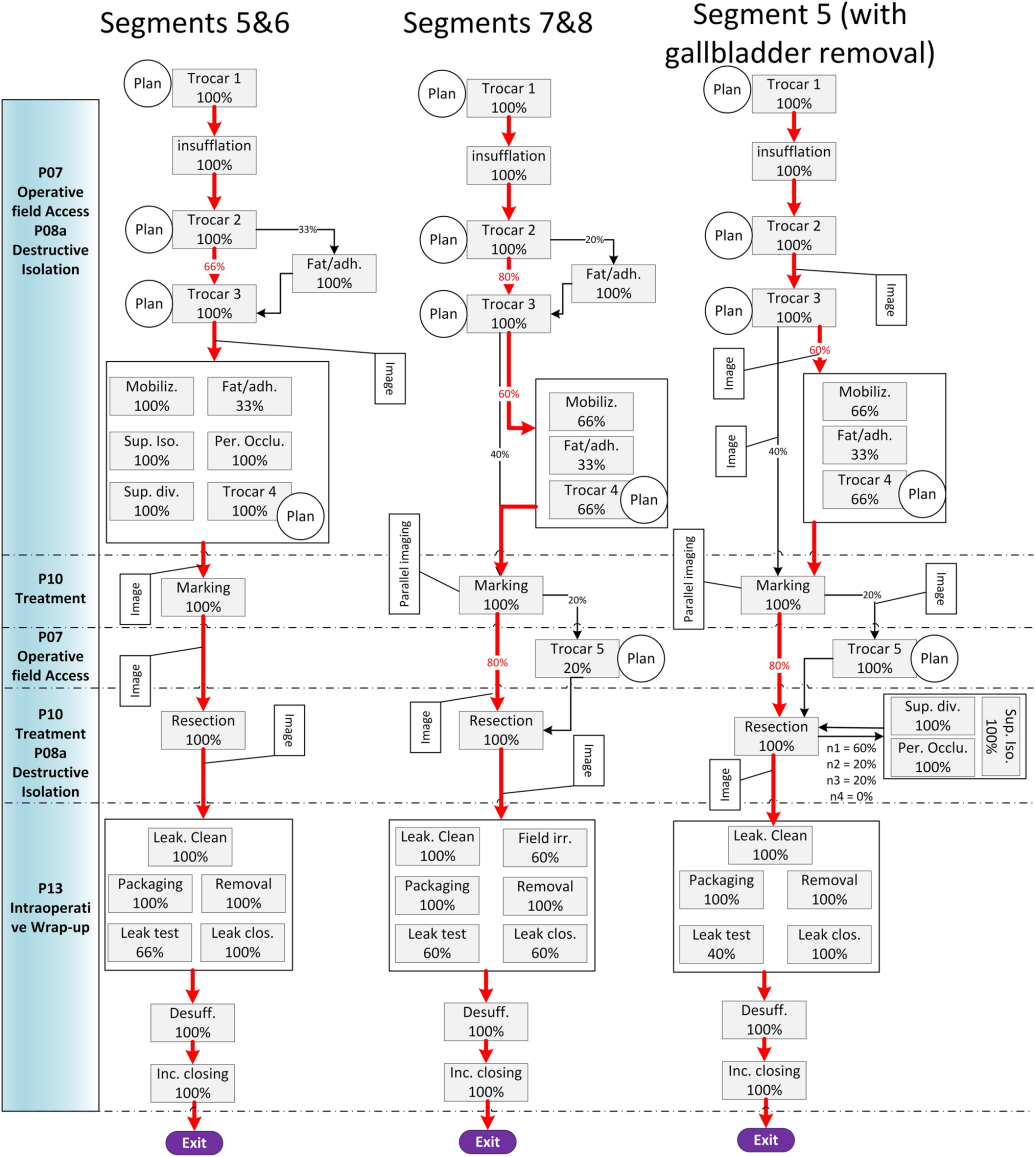
Three examples of possible paths of surgical activities in different surgery categories

**Fig. 7:**
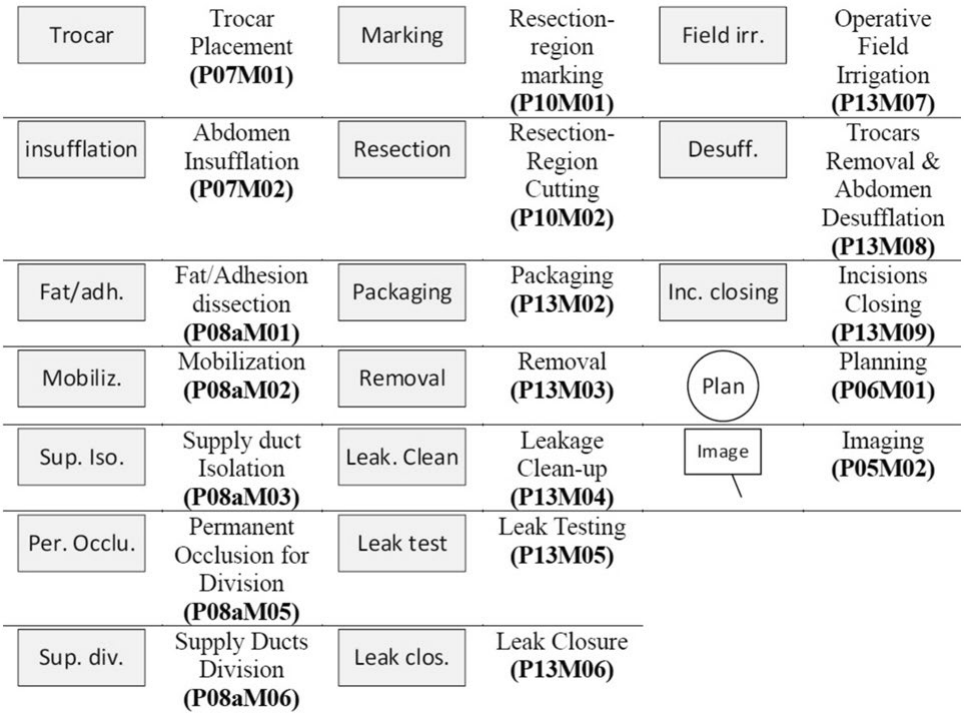
Explanation of the symbols used in Fig. 6

Figure 6 shows the possible paths of surgical activities in the three different surgery categories, see Fig. 7 for the explanation of the symbols. The most probable path for each surgery category is indicated by red arrows. The sequence of steps for each category was determined based on the data recorded for each surgery presented in [30] and https://doi.org/10.4121/20163968. All the possibilities for taking images and generating or updating plans are indicated in the figure. The rectangles show the modules and the occurrence probabilities of the modules are indicated as percentages in the rectangles. The boxes group modules that can occur in any preferred sequence. If the flow goes into one of the boxes, any and several modules can occur successively. In surgeries in Segments 5 & 6, the surgeon may isolate and divide several ducts during treatment. In this case, *n* indicates the number of occurrences; as the number of occurrence increases the probability of dividing yet another duct decreases.

To illustrate the differences between different surgery categories, the possible surgical actions for each surgery category are shown in Table 2. The table is based on analysis of data recorded for each surgery presented in [30] and https://doi.org/10.4121/20163968. The percentage in parenthesis show the probability of that module occurring once or more in a surgery. Imaging and planning are done for every surgery category and can happen anytime during the course of a surgery. Therefore, imaging and planning activities are not presented in Table 2.

**Table 2:** The workflow of different surgery categories

Phase name	Segments 5 & 6	Segments 7 & 8 (%)	Segment 5 (with gallbladder removal)
Operative field access (07)	Trocar 1 (100%)	Trocar 1 (100)	Trocar 1 (100%)
	Insufflation (100%)	Insufflation (100)	Insufflation (100%)
	Trocar 2 (100%)	Trocar 2 (100)	Trocar 2 (100%)
	Trocar 3 (100%)	Trocar 3 (100)	Trocar 3 (100%)
	Trocar 4 (60%)	Trocar 4 (80)	Trocar 4 (100%)
	–	Trocar 5 (20)	–
Destructive Isolation (08a)	Fat/Adhesion* (20%)	Fat/Adhesion* (20)	Fat/Adhesion* (33%)
	–	–	Mobilization gallbladder (100%)
	–	–	Isolation (100%)
	–	–	Perm. Occlusion (100%)
	–	–	Division (100%)
	–	–	Mobilization gallbladder
	Mobilization Liver (40%)	Mobilization Liver (40)	Mobilization Liver
Treatment (10)	Marking (100%)	Marking (100)	Marking (100%)
	Resection (100%)	Resection (100)	Resection (100%)
Destructive Isolation (08a)	Isolation (60%)	–	–
	Perm. Occlusion (60%)	–	–
	Division (60%)	–	–
Treatment (10)	Resection	–	–
Wrap-up (13)	Leakage clean-up (100%)	Leakage clean-up (100)	Leakage clean-up (100%)
	Leak Testing (40%)	Leak Testing (60)	Leak Testing (66%)
	Leak Closure (100%)	Leak Closure (60)	Leak Closure (100%)
	–	Irrigation (60)	–
	Package (100%)	Package (100)	Package (100%)
	Removal (100%)	Removal (100)	Removal (100%)
	Desufflation (100%)	Desufflation (100)	Desufflation (100%)
	Incision closing (100%)	Incision closing (100)	Incision closing (100%)

The percentages show the probability for each module that it will occur (once or more) at some time in the procedure

*Might also be influenced by other factors, such as BMI and previous abdominal surgeries

The simulations showed that introduction of the navigation platform will affect the surgical process of LLR in several ways. Based on the exclusion criteria, almost 10% of the simulation data was excluded from the simulation analysis. The convergence of the simulated data was confirmed by comparing the first batch of about 45,000 runs with a second batch of 45,000; the mean values and standard deviations differed less than 0.5% between the first and second batch.

**Fig. 8:**
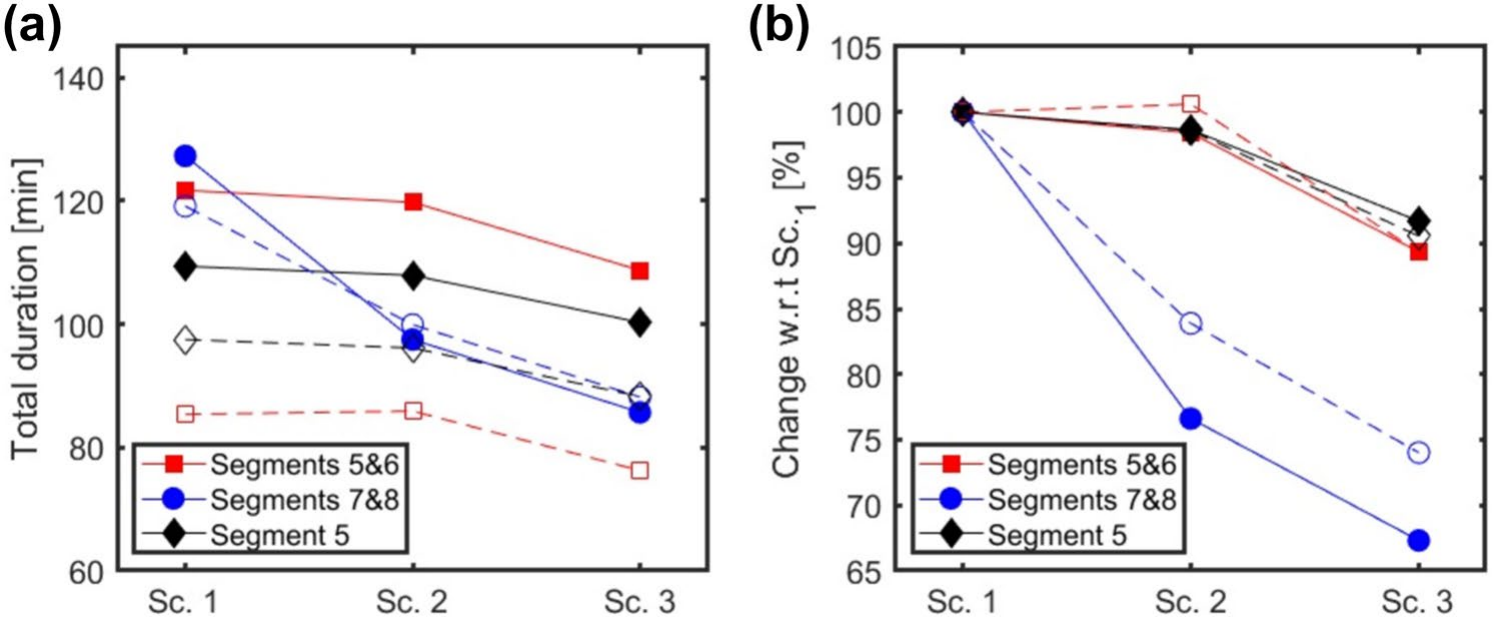
**a** The simulation results of the average of total duration of surgeries with Uniform (solid lines) and Gaussian (dashed lines) distributions. The scenarios are Sc.1 no use of the navigation platform, Sc.2 navigation platform in use—conservative positive effect, and Sc. 3 navigation platform in use—optimistic positive effect. **b** The improvements in average of total duration of surgeries in percentage with respect to scenario 1, i.e., average of total duration of Sc._x_ divided by the average of total duration of Sc._1_

Figure 8a shows the mean values of total surgery duration for all three scenarios of performing LLR. As can be seen, the choice of distribution function has a large effect on the duration of the surgeries. However, in both cases the navigation platform has a considerable effect on the total surgery duration. The simulation results show the impact of the navigation platform in different scenarios. In Scenario 3, the mean duration of surgeries decreased by 25 min compared to Scenario 1. The results in Fig. 8a and b imply that the positive impact of the navigation platform is largest for surgery on Segments 7 & 8. The improvement percentages (i.e., average of total duration of Sc._x_ (Scenario_x_) divided by the average of total duration of Sc._1_) are plotted in Fig. 8b. In Segments 7 & 8 the total duration of surgeries decreases by 20 and 30% for scenarios 2 and 3, respectively. In Segments 5 & 6 the total duration of surgeries decreases by 15% for scenario3; however, it shows a minute increase (0.6%) for scenario 2. In Segment 5, total durations decrease 2% for scenario 2 and 10% for scenario 3. A one by one analysis of modules suggests that the larger decrease in Segments 7 & 8 is due to longer imaging duration than other surgery categories.

The choice of probability distribution has a large effect (up to 30%) on the average of the total duration of surgeries, see the difference between solid line and the corresponding dashed line in Fig. 8a. Thus, predicting the true duration of surgeries depends on a reliable choice of distribution function. However, Fig. 8b suggests that the change with respect to Sc.1 of the surgeries for different scenarios only slightly depends on the choice of probability distribution function.

**Fig. 9:**
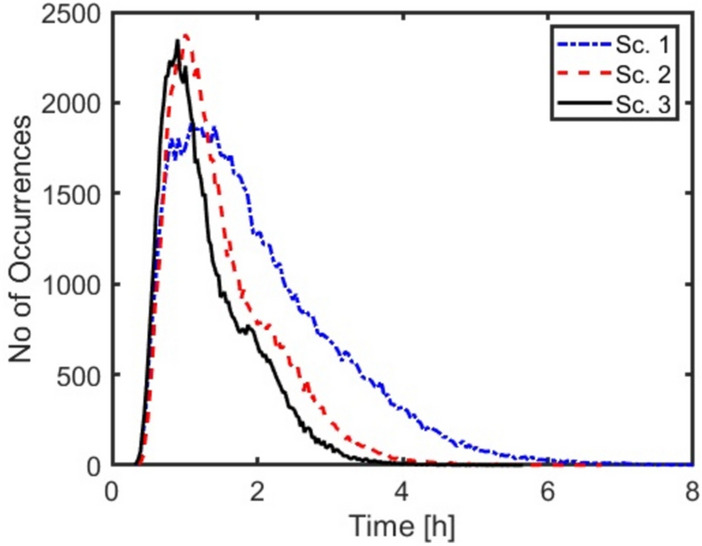
Probability distribution functions of total surgery duration of different scenarios for Segments 7 & 8 with a Uniform distribution for all modules’ durations

The probability distribution functions of total surgery duration for segments 7 & 8 are shown in Fig. 9. Based on Fig. 9, it is clear that in scenarios 2 and 3 the distribution functions are shifted toward lower values. The most probable total durations of surgeries (the peaks of the curves in Fig. 9) were decreased by 10% and 20% for scenarios 2 and 3 compared to scenario 1, respectively. The simulation data show that the potential benefit (in terms of procedure duration) of introducing new technologies depends on location of the tumor.

## Discussion

The surgical process model of LLR was analyzed for three categories of parenchyma sparing. The most probable workflow paths and the durations and occurrence frequencies of all relevant steps were presented and compared for the three surgery categories. Deriving the possible paths of treatment and the probability distribution of durations and occurrences of the surgical workflow elements out of raw surgical data enables predicting next surgical steps, improving surgical training systems, analyzing surgical performance, etc. Moreover, it provides insight into the points for improvement and bottlenecks in the surgical process.

The analyzed surgical procedures were highly variable and determining a sequence between some steps of surgical steps resulted in numerous possible surgical paths. Yet, these are covered by the flowchart in Fig. 6.

This study focused on parenchyma sparing of three tumor locations in the right lobe of the liver. In this study, we focused on the wedge resection of different segments in the right liver lobe, which is generally considered more complex than the left lobe. Specifically, we chose three categories: 7 & 8 (posterosuperior segments), 5 & 6 (anterolateral segments), and 5 (with gallbladder removal cholecystectomy). We selected data from posterosuperior segments as these are known to be extremely challenging for laparoscopy due to limited visualization, the risk of bleeding, and longer operative time. Thus, we expected the navigation platform to have a more pronounced effect on these segments (7 & 8). Moreover, we included Segments 5 & 6 and 5 to compare the results of wedge resection with and without cholecystectomy. Cholecystectomy is a relatively standard procedure, and this comparison could provide valuable insights into how the interpretation of results varies for the steps involving cholecystectomy. Overall, by examining these three categories, we aimed to gain a better understanding of the effectiveness of the navigation platform for different liver segments and procedures.

The data provided in Table 1 are based on a limited dataset of 13 interventions. However, first author MG of this paper has attended an additional 15 LLR in OUH (Oslo, Norway), Erasmus Medical Center (the Netherlands), and Bern University Hospital (Switzerland) and performed interviews with surgical teams between 2017 and 2019. These observations and interviews support that the available dataset properly represents everyday clinical practice at least in these three institutes. We made an effort to maintain consistency by selecting certain hyperparameters that could have a large impact on the procedure, such as tumor location, while keeping other factors constant (e.g., same hospital, highly skilled surgeons, malignant tumors, single lesion, right lobe). We acknowledge that a larger dataset would offer a more comprehensive analysis and account for extreme cases, but the challenges of data acquisition and availability across different medical centers, coupled with the time-consuming task of video analysis at the presented granularity level, compelled us to balance the number of analyzed videos and variation of hyperparameters. To avoid evaluating individual surgeon performance and to achieve a more generalizable interpretation of the process, we did not select only one head surgeon. Instead, we chose to analyze surgeries performed by different surgeons with similar levels of expertise. To maintain consistency, we kept the surgical teams as similar as possible by varying the head surgeons and assistants. It is worth mentioning that manual analysis and verification of endoscopic videos is a time-consuming task, consuming up to 5 days per processed surgery. Therefore, to gather more data, automated workflow steps recognition and analysis systems using artificial intelligence (AI) would be of great use. Such systems have been explored for minimally invasive surgeries, such as cholecystectomy [21], but a working automated workflow step recognition system for the level of process detail presented in this work is challenging and has, to the best of our knowledge, not yet been developed.

Automatic phase/step detection is a critical aspect of analyzing large datasets to accurately predict surgical steps during an operation. In this work, we changed one parameter (location of the tumor), while the other parameters (e.g., number of tumors, patient conditions) were kept similar. However, with automatic video analysis, it is possible to create a large dataset, to cover different variable parameters, and consequently plan and predict surgical steps, as well as the remaining time of surgery more accurately. In hybrid ORs, the data gathered from various sources is crucial for making informed surgical decisions, automating certain surgical tasks using robotic arms, and providing valuable support for surgeons to tackle the challenges posed by certain surgical cases. For instance, in LLR, changing the patient’s position can cause deformation of the liver, highlighting the need for a more precise 3D model during the operation. Analysis of surgical steps using SPM can help develop context-aware systems that can automate where intra-operative CT/ultrasound is needed to be taken for performing certain surgical steps.

Besides, SPM-based analysis of procedures and deriving possible sequences of identifiable and meaningful tasks out of highly variable surgeries aid the improvement of different aspects of the development of AI systems for automating surgical tasks. Data are the foundation for AI; however, the complexity of surgical treatments makes interpretation and management of the huge amount of data difficult. Extraction and analysis of surgical steps and the ways of performing them enable effective data acquisition, data storage, data analysis, surgical steps planning, etc. in AI systems. These capabilities contribute to the extension of existing technologies toward more autonomous surgical actions in future [44, 45].

Introduction of new technologies will affect the surgical process of LLR in several ways. A discrete event simulation model of LLR was built to investigate different scenarios that were defined for performing LLR. The changes in duration of different process model steps, as results of employing new technologies, introduced in different scenarios were estimated based on the authors’ hands-on experience with available systems. Therefore, actual performance benefits may very well deviate from the presented outcomes. It was observed that the choice of the distribution function affects the average total duration of surgeries, thus, finding a reliable distribution function is required for accurate prediction of total surgery durations. However, the compensated total duration of surgeries showed to be robust for the choice of distribution function. Nonetheless, the simulations provided much insight into what could be gained with such technology in different situations. Furthermore, the flexibility of the simulation model allows adaptation of these estimates and any other parameters in future design and optimization of new technologies for LLR. The proposed methodology has the potential to evaluate the impact of various other technologies, such robotic arms performance and surgical instrument design.

## Conclusions

The endoscopic videos from laparoscopic liver surgeries performing parenchyma sparing technique for the tumors located in Segments 5 & 6, 7 & 8, and 5 (with gallbladder removal) were analyzed to acquire detailed surgical process data. The surgeries were put into three categories based on tumor location and the most probable workflows of the surgeries in different categories were derived. In all three surgery categories, we showed that the actual treatment (P10M02) covers the major part of the total procedure duration. A discrete event simulation model was developed to predict the impact of introducing new technology. It has been shown that the impact of the proposed new navigation platform depends on the location of tumors and has the potential to decrease the surgery duration by up to 10% in Segment 5, up to 15% in Segments 5 & 6, and up to 30% in Segments 7 & 8, which is known to be difficult segments [46]. This shows the relevance of such navigation platform for difficult segments (i.e., 7 & 8), where visualization is limited. This study showed that a discrete event simulation model based on the analysis of steps during surgical procedures can be used to predict the impact of new technology.

## Data Availability

All data generated or analyzed during this study are included in this published article and its supplementary information files. The datasets generated and/or analyzed during the current study are also available in the https://doi.org/10.4121/20163968.

## Appendix

The content of this appendix is a recap from Ref. [30] for the reader’s convenience. The generic process model of MILT procedures at the highest abstraction level is shown in https://doi.org/10.4121/20163968 and Ref. [30]. The colors of the phases in the process model mean:

- Blue phases with solid-line rectangles: are shared between ablation and resection procedures. They are connected with arrows that show the flow of activities.
- Green-dashed rectangles: can happen anytime during the operation. These phases are connected to all other phases, but for the sake of readability, these arrows were left out of the figure.
- Black solid arrows: shared between ablation and resection procedures.
- Red-dashed arrows: are only for ablation procedures.
- Green-dotted-dashed arrows: the transfer of data, such as medical images and patient medical history.

The individual phases are explained below:

- Phase 01: Intake—Admission of the patient to the hospital.
- Phase 02: Pre-operative Imaging—Acquiring medical images of the patient’s liver prior to a possible operation.
- Phase 03: Pre-operative Planning—Planning regarding decisions on treatment approach, incision or needle locations, size of the target region, etc.
- Phase 04: Intra-operative Preparation—Preparation of patient, OR equipment, and surgical instruments prior to the intervention, on the day of operation.
- Phase 05: Intra-operative Imaging—Medical images can be acquired in the OR, before and during the intervention.
- Phase 06: Intra-operative Planning—Generation of update of the treatment plan in the OR just before and during intervention.
- Phase 07: Operative field Access—Making the operative field accessible by placing trocars, etc.
- Phase 08a/b: Isolation of the treatment area consists of activities to separate the target region from surrounding structures. These actions can be classified into two categories:
  - Phase 08a: Treatment Area Isolation—Destructive—Isolation by destructive (permanent) dissection, separation, or closure of surrounding structures.
  - Phase 08b: Treatment Area Isolation—Non-destructive—Isolation with temporary effects, using actions, such as temporarily closure of vessels.
- Phase 09: Needle Manipulation—Maneuvering ablation needle(s).
- Phase 10: Treatment—The actual resection or ablation of the target region.
- Phase 11: Intra-operative Complications—Managing complications that may occur during the operation.
- Phase 12: Miscellaneous—Other clinical activities might take place that do not directly serve the MILT procedure, such as biopsy and catheter placement.
- Phase 13: Intra-operative Wrap-up—All activities with the purpose of wrapping-up, such as tumor packaging and closing the incisions.

The presented process model was established through sixteen live observations of LLR, RFA, and Microwave ablations at OUH and Erasmus MC (Erasmus Medical Center, Rotterdam, The Netherlands), eight interviews with experienced surgeons at OUH and Erasmus MC, and nine offline observations of surgical procedure using endoscopic videos of laparoscopic liver treatments.

The established process model was then verified qualitatively and quantitatively. The qualitative verification was done by studying fifteen offline observation of MILT procedures from OUH, four live observations at Bern University Hospital, and two at Erasmus MC and further interviews with experienced surgeons with at least 10 years of surgical experiences in OUH and Erasmus MC. The quantitative verification is also done by analyzing fifteen offline MILT procedures from OUH. A brief description of the Modules is provided in Table 3.

**Table 3:** Different phases of generic process model of MILT and the corresponding modules according to generic surgical process model of MILT

*Phase*	Modules		Description
*Intake (01)*	*–*		All pertinent patient information is collected
*Pre-operative Imaging (02)*	*CT Imaging (1)*	P02M01	Different types of imaging modalities can provide varying levels of information regarding a patient's internal structures prior to surgery
	*US Imaging (2*	P02M02	
	*MR Imaging (3)*	P02M03	
	*FS Imaging (4)*	P02M04	
*Pre-operative Planning (03)*	*MD Meeting (1)*	P03M01	Various planning meetings with distinct objectives can be conducted before a surgical procedure. These include the multidisciplinary team meeting (M01), where different medical professionals gather to determine the most suitable treatment approach for the patient. Another important meeting is the surgical/interventional team meeting (M02), where the team discusses and coordinates the necessary equipment, instruments, and patient preparation needed for the procedure. Additionally, the lead surgeon/interventionist (M03) session is held to pre-visualize the entire surgery and all of its critical steps
	*Surg./Interv. Team Meeting (2)*	P03M02	
	*Lead Surg./Interv. Meeting (3)*	P03M03	
*Intra-operative Preparation (04)*	*Equipment Preparation (1)*	P04M01	Preparations need to be completed before the start of the operation. These preparations include equipment preparation (M01), patient preparation (M02), instrument preparation (M04), and patient positioning (M03) based on the pre-operative plan. These four modules are typically executed simultaneously
	*Patient Preparation (2)*	P04M02	
	*Patient Positioning (3)*	P04M03	
	*Instrument Preparation (4)*	P04M04	
*Intra-operative Imaging (05)*	*CT Imaging (1)*	P05M01	Different types of imaging modalities that provide different levels of information during the operation
	*US Imaging (2)*	P05M02	
	*MR Imaging (3)*	P05M03	
	*FS Imaging (4)*	P05M04	
*Intra-operative Planning (06)*	*Planning (1)*	P06M01	In the Planning module (M01), clinicians can use intra-operative images, endoscopic video, as well as data from the Pre-operative Planning module (M02) to generate or update the surgical plan based on the patient’s current condition and anatomy in the operating room In the Register Earlier Data module (M02), data from pre-operative planning and imaging are registered and made available for use in intra-operative planning
	*Register Earlier Data (2)*	P06M02	
*Operative field access (07)*	*Trocar placement (1)*	P07M01	During laparoscopic liver resection (LLR) and laparoscopic liver ablation (LLA), the surgeon needs to make the operative field accessible. This involves placing a trocar (M01) and insufflating the patient’s abdomen with carbon dioxide (M01) to obtain access to the operative field. Additionally, the surgeon may use a fixed retractor (M03) to hold the liver or surrounding organs in place
	*Abdomen Insufflation (2)*	P07M02	
	*Retractor Placement (3)*	P07M03	
*Destructive Isolation (8a)*	*Fat/adhesion Dissection (1)*	P08aM01	This phase involves three primary actions: dissection of fat and adhesions (M01), mobilization of the liver or surrounding organs (M02), and division of the supply ducts (M03, M04, M05, and M06). To safely divide the supply ducts (including hepatic artery branches, and portal veins, bile duct and hepatic veins) the surgeon may first need to isolate the ducts (M03) from surrounding tissues and structures. Prior to division, the ducts are carefully permanently occluded (M05) by clips or stapler device. The criteria for permanent occlusion is the usage of clips or stapler device. In some cases, temporary occlusion of the supply ducts (M04) may be necessary to confirm the location and closure of the target vessels (usually in formal/major resections). Once the supply ducts are confirmed and occluded, they can be divided (M06)
	*Organ Mobilization (2)*	P08aM02	
	*Supply Ducts Isolation (3)*	P08aM03	
	*Temporary Occlusion for Division (4)*	P08aM04	
	*Permanent Occlusion for Division (5)*	P08aM05	
	*Supply Ducts Division (6)*	P08aM06	
*Treatment Area Isolation – Non-destructive (8b)*	*Vessels Isolation (1)*	P08bM01	This phase consists of two categories of actions. For laparoscopic procedures the surgeon first isolates any relevant vessels (M01) and then occludes them temporarily (M02) to reduce bleeding during treatment of the target region, such as by performing the Pringle maneuver
	*Temporary Occlusion Application (2)*	P08bM02	
	*Artificial Fluid Injection (3)*	P08bM03	
*Treatment (10)*	*Region Marking (1)*	P10M01	In the case of LLR, the surgeon needs to determine the resection margins and might need to mark (M01) physically on the organ (common in case of parenchyma sparing resection). After this, the surgeon can proceed with cutting the resection region (M02)
	*Resection Region Treatment (2)*	P10M02	
	*Target Region Ablation (3)*	P10M03	
*Intra-operative complications (11)*	*Surgical Drainage (1)*	P11M01	Complications may arise during the operation, and in order to manage these situations, different actions may need to be taken. For example, the surgeon may need to place surgical drainage (M01) or administer a blood transfusion (M02). In the case of damage to internal structures, repair procedures (M04) may need to be carried out. Additionally, if there is any leakage from the damaged structures, the surgeon may need to clean it up (M03)
	*Leakage Clean-up (2)*	P11M02	
	*Blood Transfusion (3)*	P11M03	
	*Repair Damaged Structures (4)*	P11M04	
*Miscellaneous (12)*	*Chemo Catheter Insertion (1)*	P12M01	During the operation, various additional activities may be performed that are not directly related to MILT, such as inserting a catheter into a vessel (M01) to deliver chemotherapy medications or performing a liver biopsy (M02) for further examination purposes
	*Liver Biopsy (2)*	P12M02	
*Wrap-up (13)*	*Needle Removal (1)*	P13M01	After the treatment, the surgeon/interventionist needs to properly close the operative field. This includes removing the ablation needle (M01), disposing of any waste materials (M02 and M03), controlling and cleaning up any leakage (M04, M05, M06, and M07), desufflating the abdomen, and closing the incision (M08 and M09)
	*Packaging (2)*	P13M02	
	*Removal (3)*	P13M03	
	*Leakage Clean-up (4)*	P13M04	
	*Leak Testing (5)*	P13M05	
	*Leak Closure (6)*	P13M06	
	*Operative Field Irrigation (7)*	P13M07	
	*Trocars Removal & Abdomen Desufflation (8)*	P13M08	
	*Incisions Closing (9)*	P13M09	
